# Editorial: Neuroimmune cell signaling in COVID-19

**DOI:** 10.3389/fimmu.2024.1429908

**Published:** 2024-05-23

**Authors:** Kiarash Saleki, Zahra Mojtahedi, Timo Ulrichs, Mehdi Mahdavi, Abbas Azadmehr

**Affiliations:** ^1^ Student Research Committee, Babol University of Medical Sciences, Babol, Iran; ^2^ USERN Office, Babol University of Medical Sciences, Babol, Iran; ^3^ Department of E-Learning in Medical Sciences, Faculty of Medical Education and Learning Technologies, Shahid Beheshti University of Medical Sciences, Tehran, Iran; ^4^ Northern Arizona University, Flagstaff, AZ, United States; ^5^ Institute for Research in International Assistance, Akkon University for Human Sciences, Berlin, Germany; ^6^ Advanced Therapy Medicinal Product (ATMP) Department, Breast Cancer Research Center, Motamed Cancer Institute, Academic Center for Education, Culture and Research (ACECR), Tehran, Iran; ^7^ Department of Immunology, Babol University of Medical Sciences, Babol, Iran

**Keywords:** COVID-19, neuroimmunology, microglia, Guillain Barre syndrome (GBS), multiple sclerosis, Toll-like receptor (TLR), inflammasome

The Coronavirus disease-2019 (COVID-19) pandemic started in Wuhan, China, and has ever since taken the world by storm. COVID-19 was caused by severe acute respiratory syndrome coronavirus 2 (SARS-CoV-2), a member of the human coronavirus family responsible for two previous outbreaks. SARS-CoV2 spread rapidly around the globe, resulting in death in large numbers (1–3). A large retrospective cohort showed that the approximate rate of a neurological or psychiatric event after COVID-19 was one in three cases or one in eight cases in which a neurological or psychiatric condition was diagnosed for the first time. These epidemiological rates reach more than 45% of critically ill ICU COVID-19 cases (4).

SARS-CoV2 has been suggested to enter the central nervous system (CNS) via a retrograde non-hematogenous route through the olfactory bulb and by utilizing angiotensin-converting enzyme 2 (ACE2) (1, 2). More recent research has established neuropilin (NRP) 1 as an entry factor for COVID-19 that accelerates neuroinvasion by SARS-CoV2 within the central or peripheral nervous system. In addition, NRPs play a major role in the hyperinflammatory injury of the lung, nervous system, kidneys, liver, pancreas, and heart in COVID-19 cases. These effects result from interaction with specific regions of the SARS-CoV2 spike protein, and vascular endothelial growth factor (VEGF) receptors (VEGFR1/2) (5, 6), highlighting the role of multi-organ neuroimmunopathological injury in COVID-19.

When immune system Toll-like receptors (TLRs) are exposed to SARS-CoV2 viral RNA and protein elements, hyperinflammation develops in multiple organs, including the lungs and CNS (7, 8). Uncontrolled hyperinflammation in COVID-19 patients leads to a dysregulated immune response in the body leading to multi-organ neuroimmunological injury (9–11).

On the other hand, our *Frontiers in Immunology* Research Topic has shown that the COVID-19 vaccination influences the immune response. Neurological adverse events following the COVID-19 vaccination have been identified such as Guillain-Barré Syndrome (GBS). Novel mechanisms for the efficacy of vaccination in patients with pre-existing neurological conditions such as multiple sclerosis (MS) have also been reported (Nytrova et al.).

The present Research Topic includes a variety of works addressing neuroimmunity in COVID-19, innate or adaptive immune dysregulations caused by COVID-19, and therapeutics that target neuroimmune mechanisms in COVID-19.

Santos Alves et al. demonstrated that the SARS-CoV2 S protein mediates microglial purinergic pathways unraveling new avenues to explore the role of purinergic receptors in the control of COVID-19 adverse events. Moreover, they showed by immunohistochemistry experiments that the expression of the purinergic P2X7 receptor was increased in microglia in specific hippocampal regions after spike infusion. Their findings suggest that the P2X7 receptor modulates innate and adaptive immune responses associated with activation of TLR4, nuclear factor kappa light chain enhancer of B-cell (NF-κB), and inflammasomes, leading to neuroinflammatory and chronic stress conditions (12–19).

In addition, the role of vaccination in patients with neurodegenerative diseases was presented in this Research Topic, with a special focus on MS. Scientists worldwide agree that the potential benefit of COVID-19 vaccination in MS patients is likely to be much higher than the side effects of vaccination (Pugliatti et al.). In the same context, Räuber et al. conducted an original investigation and found that more COVID-19 vaccinations may improve the humoral immunological feedback in Ocrelizumab (anti-CD20)-treated MS cases and enhance clinical protection against COVID-19. Their findings suggest that the vaccines efficiently confer protection even in Ocrelizumab-treated MS patients without detectable COVID-19-specific humoral immunological feedback, implying that compensatory processes may be at play, such as T-lymphocyte-regulated pathways.

Another article from our Research Topic showed that the majority of cases of MS, myasthenia gravis (MG), and neuromyelitis optica spectrum disorders (NMOSD) developed a SARS-CoV2-exclusive T-lymphocyte response. This information may justify how vaccination can activate the production of SARS-CoV2-exclusive antibodies in a fraction of anti-CD20- administered cases. Furthermore, the seroconversion ratio was increased in Ocrelizumab-administered cases in comparison with Rituximab (RTX) (Nytrova et al.). In this context, Groß-Albenhausen et al. suggested optimal vaccination intervals for Ocrelizumab-treated MS patients.

Also in the present topic, Bellucci et al. showed in a case report that a possible adverse effect of COVID-19 vaccination could be the development of GBS. The authors hypothesized that molecular mimicry or anti-idiotype antibodies could be the processes responsible for such adverse neurological effects. It is recommended that future studies evaluate the suitability of COVID-19 revaccination in cases with previous para-COVID-19 or post-COVID-19 GBS patients who are serologically positive for ganglioside antibodies. In line with this, this Research Topic presents a review by Yu et al. on the topic of GBS and COVID-19.

In summary, this collection of articles suggests that in COVID-19 patients with autoimmune diseases such as MS, vaccination either protects against SARS-CoV2 infection or the dosage and timing of immunization should be cautiously optimized, in order to maximize efficacy and minimize adverse events associated with vaccination. Additionally, other works on this Research Topic have indicated that COVID-19 vaccination may be associated with the development of autoimmune conditions such as GBS.

We emphasize that, beyond the scope of this Research Topic, many published reports have shown that COVID-19 vaccination may have affected the prevalence and development of autoimmune diseases through molecular mimicry or neutrophil extracellular trap (NETosis), and also cancer in particular through molecular mechanisms involving the SARS-CoV2 entry and the angiogenesis factor NRP-1 post-vaccination. These reports suggest that more studies are warranted in different patient subpopulations to characterize specific side effects following COVID-19 vaccination.

## Author contributions

KS: Conceptualization, Data curation, Formal analysis, Funding acquisition, Investigation, Methodology, Project administration, Resources, Software, Supervision, Validation, Visualization, Writing – original draft, Writing – review & editing. ZM: Writing – original draft, Writing – review & editing. TU: Writing – original draft, Writing – review & editing. MM: Writing – original draft, Writing – review & editing. AA: Conceptualization, Data curation, Formal analysis, Funding acquisition, Investigation, Methodology, Project administration, Resources, Software, Supervision, Validation, Visualization, Writing – original draft, Writing – review & editing.
